# Alteration of Extracellular Matrix Molecules and Perineuronal Nets in the Hippocampus of Pentylenetetrazol-Kindled Mice

**DOI:** 10.1155/2019/8924634

**Published:** 2019-11-14

**Authors:** Hiroshi Ueno, Shunsuke Suemitsu, Shinji Murakami, Naoya Kitamura, Kenta Wani, Yu Takahashi, Yosuke Matsumoto, Motoi Okamoto, Takeshi Ishihara

**Affiliations:** ^1^Department of Medical Technology, Kawasaki University of Medical Welfare, Okayama 701-0193, Japan; ^2^Department of Psychiatry, Kawasaki Medical School, Okayama 701-0192, Japan; ^3^Department of Neuropsychiatry, Graduate School of Medicine, Dentistry and Pharmaceutical Sciences, Okayama University, Okayama 700-8558, Japan; ^4^Department of Medical Technology, Graduate School of Health Sciences, Okayama University, Okayama 700-8558, Japan

## Abstract

The pathophysiological processes leading to epilepsy are poorly understood. Understanding the molecular and cellular mechanisms involved in the onset of epilepsy is crucial for drug development. Epileptogenicity is thought to be associated with changes in synaptic plasticity; however, whether extracellular matrix molecules—known regulators of synaptic plasticity—are altered during epileptogenesis is unknown. To test this, we used a pentylenetetrazole- (PTZ-) kindling model mouse to investigate changes to hippocampal parvalbumin- (PV-) positive neurons, extracellular matrix molecules, and perineuronal nets (PNNs) after the last kindled seizure. We found an increase in Wisteria floribunda agglutinin- (WFA-) and Cat-315-positive PNNs and a decrease in PV-positive neurons not surrounded by PNNs, in the hippocampus of PTZ-kindled mice compared to control mice. Furthermore, the expression of WFA- and Cat-315-positive molecules increased in the extracellular space of PTZ-kindled mice. In addition, consistent with previous studies, astrocytes were activated in PTZ-kindled mice. We propose that the increase in PNNs after kindling decreases neuroplasticity in the hippocampus and helps maintain the neural circuit for recurrent seizures. This study shows that possibility of changes in extracellular matrix molecules due to astrocyte activation is associated with epilepticus in PTZ-kindled mice.

## 1. Introduction

Epilepsy is one of the most common chronic neurological disorders with a prevalence of 0.4% to 1.0% [1]. To date, none of the drugs used to treat epilepsy are able to prevent its onset or reverse epileptogenesis [2]. Therefore, the current treatment of epilepsy merely prevents or suppresses seizures, rather than halting the process of developing epilepsy. The pathophysiological processes leading to epilepsy are poorly understood. A better understanding of the molecular and cellular mechanisms involved in the onset of epilepsy will help the development of therapeutic agents that alter or halt epileptogenesis.

Kindling is an experimental epilepsy model, where repeated electrical or chemical stimulation of certain forebrain structures gradually causes stronger signals on electroencephalography and behavioral seizure activity [3, 4]. Once established, kindling results in a permanent state of seizure susceptibility.

Although seizures can induce neuronal death, they also have nonfatal pathophysiological effects on neuronal structure and function [5]. For example, epileptogenesis is associated with changes to synaptic plasticity [6, 7]. However, what causes and maintains these changes has not been clarified. The extracellular matrix (ECM)—which fills the extracellular domain of all organs and tissues and contains water and ions [8, 9]—is thought to regulate synaptic plasticity; whether it is altered in the early stages of epilepsy is currently unknown.

ECM in the central nervous system (CNS) is formed from hyaluronic acid, tenescin-R, glycoproteins, and chondroitin sulfate proteoglycans (CSPGs) [8]. In the mature CNS, ECM molecules are distributed as neural granules or perineuronal nets (PNN) [8]. A PNN is a mesh-like special structure that covers the cell body, axonal origin, and proximal dendrites of specific neurons in the CNS [10]. In the cortex and hippocampus, PNNs mainly form around parvalbumin- (PV-) positive GABAergic neurons [10]. Although the exact function of PNNs is not known, they stabilize synaptic connections, regulate synaptic plasticity, participate in ECM linkage with the cytoskeleton, and promote neuron-astrocyte interactions [11–13]. PNNs form late in development, and are thought to reduce synaptic plasticity leading to the termination of the critical period of the CNS. Indeed, it has been suggested that aggrecan, a component of PNNs, controls plasticity [14–16]. Once mature synapses are established and surrounded by aggrecan-containing PNNs, they are stable and poorly reorganized in adults [14, 17].

The vegetable lectin Wisteria floribunda agglutinin (WFA) is widely used to detect PNNs. WFA binds to N-acetylgalactosamine [18–20]. The antibody Cat-315 against aggrecan is also often used to detect PNNs. Cat-315 recognizes HNK-1 carbohydrate epitope of aggrecan [14, 21, 22]. In this study, WFA lectin and Cat-315 were used to investigate the expression of extracellular matrix molecules and PNNs.

About 80% of parvalbumin- (PV-) positive GABAergic interneurons are surrounded by PNNs ([23]. GABAergic inhibitory interneurons are known to regulate excitatory neurons and play an important role in epileptogenesis by causing an imbalance in excitation and inhibition [24–26]. GABAergic interneurons can be classified into various subclasses according to their anatomical, neurochemical, and electrophysiological characteristics [27–30]. PV-expressing fast-spiking (100-800 Hz) interneurons account for 40-50% of GABAergic interneurons and form synapses at the soma and axon initial segment of pyramidal cells [31, 32]. PV-positive neurons generate strong feedforward inhibition [33, 34]. PV-expressing basket cells extend their axons to the somata of pyramidal neurons. In addition, PV-expressing axo-axonic cells innervate the axon initial segments of pyramidal neurons. In the hippocampus, the vast majority of PV-expressing basket cells were surrounded by WFA-positive PNNs, while PV-expressing axo-axonic cells often lacked WFA-positive PNNs [35].

Astrocytes—a group of specialized glial cells in the CNS that control ion concentrations, neurotransmitter homeostasis, metabolism, and synapse development and signaling [36]—are one of the major sources of both ECM components and modulators. During epileptic seizures, astrocytes respond rapidly by exhibiting a form and function change called reactive astrogliosis [37]. Whether reactive astrogliosis following seizures results in ECM remodeling—which plays a role in synaptic plasticity both during postnatal development and after injury—is currently unclear. Astrocytes control synaptic plasticity in glutamatergic synapses by releasing of *D*-serine [38]. Previous study has shown that the reduced Ca^2+^-dependent release of *D*-serine by astrocytes impairs initiation of synaptic plasticity [39]. Therefore, astrocyte can potentially play an important role in epileptogenesis.

The purpose of this study is to determine whether early-onset seizure activity is associated with changes to ECM molecules and astrocytes in an experimental model of epilepsy. Increasing our understanding of ECM and astrocyte changes in vivo following an increase in seizure activity will help reveal the specific mechanistic role of astrocytes in epilepsy. The results obtained in this study may provide an opportunity to develop new therapeutic approaches to prevent seizures or their consequences.

## 2. Material and Methods

### 2.1. Animals

Eleven-week-old male mice (C57BL/6N) were used for experiments. Mice were housed five to a cage under standard laboratory conditions. All procedures related to animal maintenance and experimentation were approved by the Committee for Animal Experiments at Kawasaki Medical School Advanced Research Center and conformed to the U.S. National Institutes of Health (NIH) Guide for the Care and Use of Laboratory Animals (NIH Publication No. 80-23, revised in 1996). We purchased the mice from Charles River Laboratories (Kanagawa, Japan). The mice were housed in a room with a 12-hour light/dark cycle (light on 8:00 a.m. and off 8:00 p.m.) with temperature maintained at 23–26°C. They were provided with nesting material, food, and water *ad libitum*.

### 2.2. Pentylenetetrazole Kindling Procedure

All mice were randomized into two groups (*n* = 15). Pentylenetetrazole (PTZ; Sigma-Aldrich Japan, Tokyo, Japan) was dissolved in saline to prepare concentrations of 20 mg/mL. A dose of 40 mg/kg was injected intraperitoneal for a total period of 15 days. Vehicle control mice were injected with saline. Seizure events during a 30 min period after each PTZ injection were observed. The resultant seizures were scored as follows [40]: stage 0 (no response), stage 1 (ear and facial twitching), stage 2 (myoclonic body jerks), stage 3 (forelimb clonus, rearing), stage 4 (clonic convulsions, turn onto the side), and stage 5 (generalized clonic convulsions, turn onto the back). On day 16, mice were sacrificed and the brains were removed.

### 2.3. Tissue Preparation

We did following methods in Ueno et al. [15, 41]. Mice were anesthetized with a lethal dose of sodium pentobarbital (120 mg/kg, i.p.) and transcardially perfused with 25 mL of phosphate-buffered saline (PBS) followed by 100 mL of 4% paraformaldehyde in PBS (pH 7.4). Brains were dissected and postfixed overnight at 4°C in the above fixative. The brains were then cryoprotected in 15% sucrose for 12 h followed by 30% sucrose for 20 h at 4°C. Next, the brains were frozen in an optimum cutting temperature compound (Tissue-Tek; Sakura Finetek, Tokyo, Japan) using a slurry of normal hexane in dry ice. Serial coronal sections with a thickness of 40 *μ*m were obtained at -20°C using a cryostat (CM3050S; Leica Wetzlar, Germany). The sections were collected in ice-cold PBS containing 0.05% sodium azide.

### 2.4. Immunohistochemistry

We treated the cryostat sections with 0.1% Triton X-100 with PBS at room temperature for 15 min. After three washes with PBS, we incubated the sections with 10% normal goat serum (ImmunoBioScience Corp., Mukilteo, WA) in PBS at room temperature for 1 h, we washed them three times with PBS, and we incubated them overnight at 4°C in PBS containing biotinylated WFA (B-1355, Vector Laboratories; 1 : 200) and/or the antibodies described in Antibodies and Lectins. After washing with PBS, we incubated the sections with Alexa Fluor 594-conjugated streptavidin (S11227; Molecular Probes, Eugene, OR) and/or the corresponding secondary antibodies (described in Antibodies and Lectins) at room temperature for 2 h. We rinsed the labeled sections again with PBS, and we mounted them on glass slides with VECTASHIELD medium (H-1400; Vector Laboratories, Funakoshi Co., Tokyo, Japan). We stored the prepared slides at 4°C until we used them in the microscopy analysis.

### 2.5. Antibodies and Lectins

We used the following lectins and primary antibodies for staining: biotinylated WFA (B-1355, Vector Laboratories; 1 : 200), mouse anti-parvalbumin (clone PARV-19, P3088; Sigma-Aldrich Japan, Tokyo, Japan; 1 : 1,000), mouse anti-NeuN (clone A60, MAB377; Millipore, Bedford, MA; 1 : 500), mouse anti-aggrecan (Cat-315; MAB1581, Millipore; 1 : 1,000), rabbit anti-GFAP (ab7260; Abcam, Cambridge, MA; 1 : 1,000), rabbit anti-IBA-1 (019-19741; FUJIFILM Wako Pure Chemical Corporation, Osaka, Japan; 1 : 1,000), mouse anti-GAD67 (clone 1G10.2, MAB5406; Millipore; 1 : 1,000), and guinea pig anti-VGLUT1 (AB5905; Millipore; 1 : 1,000).

We used the following secondary antibodies for visualization: Alexa Fluor 488-conjugated goat anti-mouse IgG (ab150113; Abcam, Cambridge, MA; 1 : 1,000), Alexa Fluor 594-conjugated goat anti-guinea pig (A-11076; Thermo Fisher Scientific, Waltham, MA; 1 : 500), FITC-conjugated anti-mouse IgM (sc-2082, Santa Cruz Biotechnology, Santa Cruz, CA, 1 : 1,000), Texas Red-conjugated goat anti-rabbit (TI-1000; Vector Laboratories, Funakoshi Co., Tokyo, Japan), and streptavidin-conjugated Alexa Fluor 594 (S11227, Thermo Fisher Scientific; 1 : 1,000).

### 2.6. Microscopy Imaging

To quantify the density of PV- and WFA-positive PNNs and analysis of ECM fluorescence intensity, we used a confocal laser scanning microscope (LSM700; Carl Zeiss, Oberkochen, Germany) to obtain images of stained sections. Images (1024 × 1024 pixels) were saved as TIFF files using the ZEN software (Carl Zeiss). Briefly, we performed the analysis using a 10x objective lens and a pinhole setting that corresponded to a focal plane thickness of less than 1 *μ*m. For observing ECM molecules and GAD67- and VGLUT1-positive synaptic terminals, samples were randomly selected and high-magnification images using a 100x objective lens were acquired. Prior to capture, the exposure time, gain, and offset were carefully set to ensure a strong signal but to avoid saturation. Identical capture conditions were used for all sections. Images from whole sections were acquired using a 10x objective lens of a fluorescence microscope (BZ-X; KEYENCE, Tokyo, Japan), and we merged them using the KEYENCE BZ-X Analyzer software (KEYENCE).

### 2.7. Quantification of Labeled PNNs and ECM Molecules

Brain regions were determined in accordance with the mouse brain atlas of Paxinos and Franklin [42]. From each mouse, four serial coronal dorsal hippocampal sections (from -0.8 mm to -1.8 mm relative to bregma) were selected and processed for staining. All confocal images were acquired as TIFF files and analyzed with the NIH ImageJ software (NIH, Bethesda, MD; http://rsb.info.nih.gov/nih-image/). Stained PNNs (soma size above 60 *μ*m^2^) were manually tagged and counted within the area of interest. Background intensity was subtracted using unstained portions of each section. Labeled neuron density was calculated as cells/mm^2^. Quantifications were performed by a blinded independent observer. To quantify the fluorescence intensity of ECM-positive molecules, GAD67- and VGLUT1-positive synaptic terminals, WFA- and Cat-315-positive PNNs, we selected four sections from each mouse brain and stained them as described in Sections 2.4 and 2.5. The ellipse circumscribing the WFA- and Cat-315-positive PNNs was traced manually on 8-bit images of each section, and the gray levels for WFA or Cat-315 labeling were measured using the ImageJ software, which was assigned arbitrary units (a.u.). We manually outlined the parts excluding PNNs and measured the gray level with NIH ImageJ. Background intensity was subtracted using unstained portions of each section. We acquired all confocal images as TIFF files and analyzed them with NIH ImageJ. We coded the slides, and a blinded independent observer quantified them.

### 2.8. Data Analysis

Statistical analysis was conducted using the SPSS software (IBM Corp., Tokyo, Japan). To test for the area and PTZ kindling effects, statistical significance was determined by two-way analysis of variance followed by Bonferroni *t*-tests. The statistical significance threshold was set at *p* < 0.05. Data are expressed as the mean ± SEM of 6 animals per group.

## 3. Results

### 3.1. PTZ-Kindled Mouse Model

**Figure 1:**
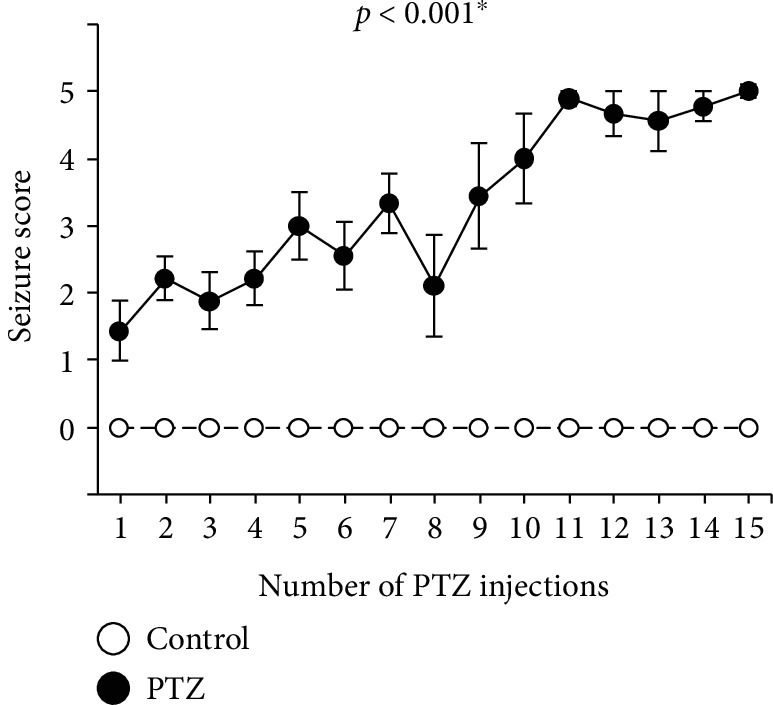
The pentylenetetrazole-kindled model mice were administered PTZ 15 times at a dose of 40 mg/kg. Data are expressed as mean ± standard error of the mean (SEM). ^∗^*p* < 0.05 versus saline-treated mice. The *p* values were calculated using repeated measures ANOVA. PTZ: pentylenetetrazole.

The PTZ-kindled mouse model was generated by treating the mice with PTZ at a dose of 40 mg/kg. The mice repeatedly administered PTZ showed a progressive development of seizures, compared to control mice (Figure 1; *F* = 5163.6, *p* < 0.001). Of 15 mice used in the present experiments, 10 mice reached a kindled status for a total period of 15 days. There was a greater seizure-associated mortality in the PTZ-kindled mouse. Nine of the 15 PTZ-kindled mice died before the end of the experiment. No control mice died unexpectedly.

**Figure 2:**
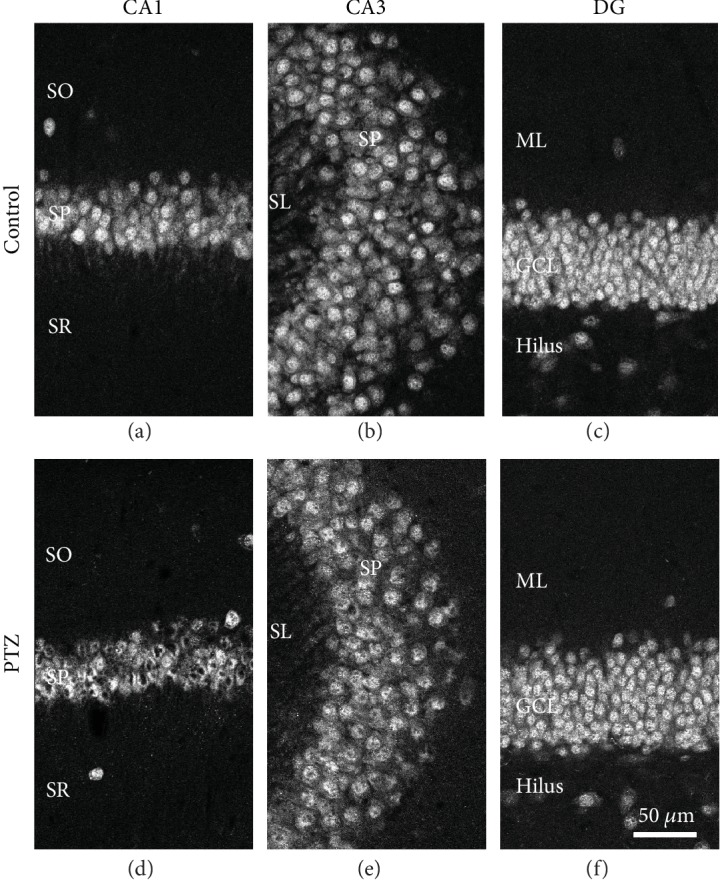
NeuN-positive images in the PTZ-kindled mouse hippocampus. Representative NeuN-positive images of the CA1 (a, d), CA3 (b, e), and DG (c, f) in control (a–c) and PTZ-kindled mice (d–f) are shown. Scale bar: 50 *μ*m. PTZ: pentylenetetrazole.

To examine whether neurodegeneration occurred following seizures, we first looked for changes to NeuN-positive cells in the hippocampus in fully kindled mice (Figure 2(a)–(f)). While there were no apparent changes in the distribution of NeuN-positive cells in the hippocampus of PTZ-kindled mice, NeuN-positive cells showed nuclear anomaly compared with controls, indicative of neurodegeneration. Perturbed nucleoli were observed in the hippocampus of PTZ-kindled mice (Figure 2(d) and (e)).

### 3.2. PV-Positive Neurons, WFA-Positive, and Cat-315-Positive PNNs in the PTZ-Kindled Mouse Hippocampus

**Figure 3:**
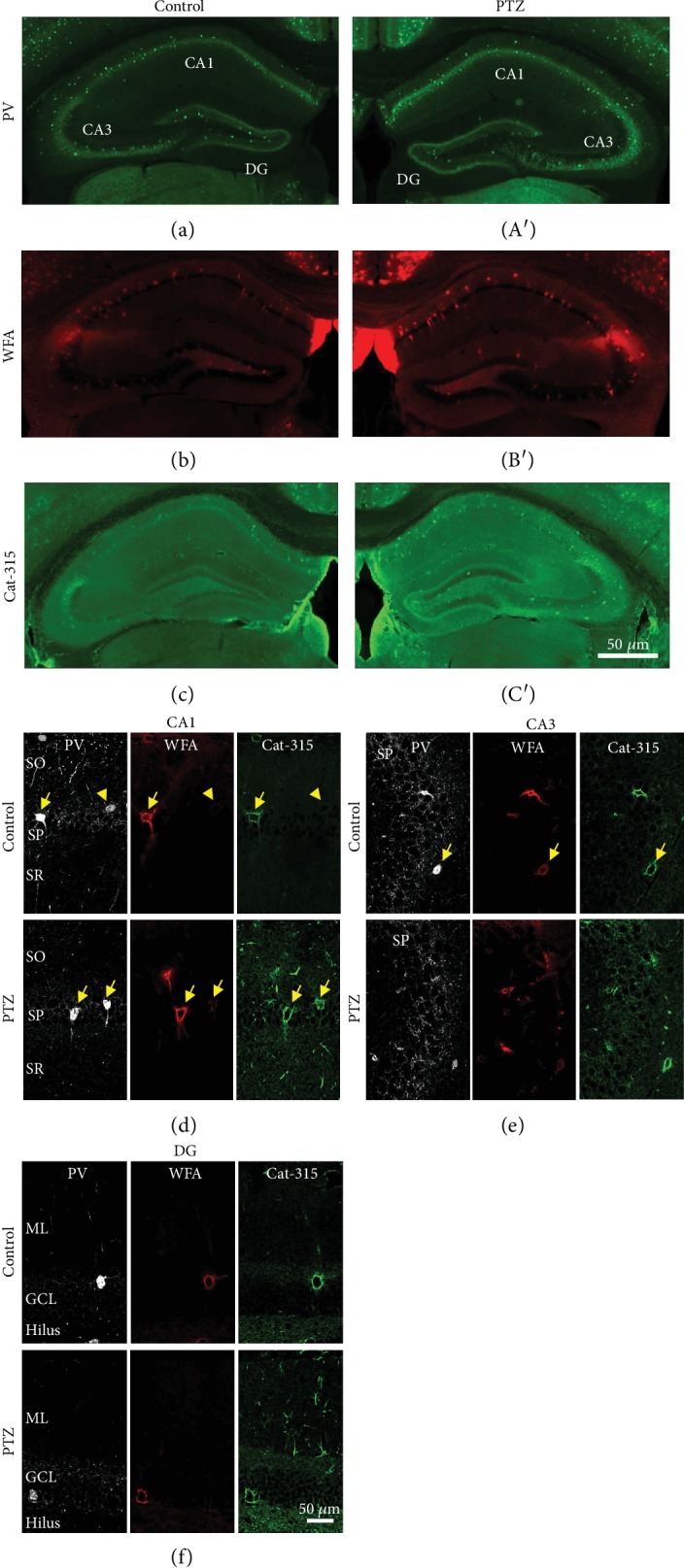
PV, WFA, and Cat-315-positive images in the PTZ-kindled mouse hippocampus. Representative images show the laminar distribution of PV-positive neurons (a, A′), WFA-positive molecules (b, B′), and Cat-315-positive molecules in the mouse hippocampus of control (a–c) and PTZ-kindled mice (A′–C′). Representative triple immunofluorescent images show the laminar distribution of PV neurons, WFA-positive PNNs, and Cat-315-positive PNNs in the CA1 (d), CA3 (e), and DG (f) of control (upper panels) and PTZ-kindled mice (lower panels). The arrows indicate PV-positive neurons colocalized with PNN markers. The arrowheads indicate PV-positive neurons not colocalized with PNN markers. Scale bars: 500 *μ*m in C′ (applies to (a)–(c), (A′)–(C′); 50 *μ*m in (f) (applies to (d)–(f)). PV: parvalbumin; WFA: Wisteria floribunda agglutinin; PNN: perineuronal nets; PTZ: pentylenetetrazole.

PV-positive neurons were observed in the hippocampus of PTZ-kindled mice (Figures 3(a), (A′), and 3(d)–(f)). To examine the spatial distribution of PNNs and ECM molecules, we stained for WFA and Cat-315, which detects aggrecan (Figure 3(b)–(f)). The distribution of PV-positive neurons, WFA- and Cat-315-positive PNNs in the hippocampus were very similar in control and PTZ-kindled mice. An enlarged image of Cat-315-positive molecules under the same conditions is shown in Figure 3(d)–(f), revealing that Cat-315 fluorescence intensity differed between control and PTZ-kindled mice.

**Figure 4:**
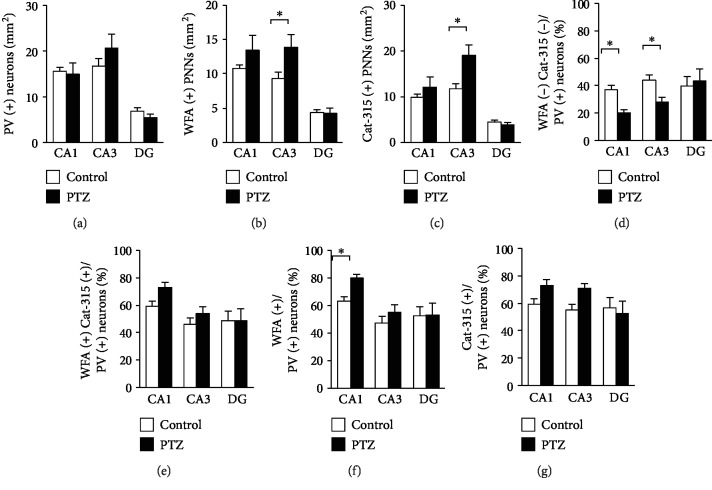
The densities of PV-positive neurons and WFA- and Cat-315-positive PNNs in the PTZ-kindled mouse hippocampus. Region-specific patterns of PV neuron density (a), WFA-positive PNN density (b), and Cat-315-positive PNN density (c) are shown. The percentage of PV-positive neurons that do not contain both WFA- and Cat-315-positive PNNs (d), the percentage of PV-positive neurons that contain both WFA- and Cat-315-positive PNNs (e), the percentage of PV-positive neurons that contain WFA-positive PNNs (f), and the percentage of PV-positive neurons that contain Cat-315-positive PNNs (g) in the hippocampus are shown. All data are presented as the mean ± SEM (*n* = 6 mice per group). The *p* values indicate two-way ANOVA followed by *post hoc* Bonferroni *t*-tests. ^∗^*p* < 0.05. P: parvalbumin; WFA: Wisteria floribunda agglutinin; PNN: perineuronal nets; PTZ: pentylenetetrazole.

We quantified PV-positive neurons and WFA- and Cat-315-positive PNNs in the PTZ-kindled mouse hippocampus (Figures 4(a)–4(c)). While there was no difference in the density of PV-positive neurons in all three areas (CA1, CA3, and DG) (Figure 4(a); *F*_2,70_ = 1.491, CA1: *p* = 0.803, CA3: *p* = 0.097, and DG: *p* = 0.561), the density of both WFA- and Cat-315-positive PNNs was significantly higher only in the CA3 region of PTZ-kindled mice compared with control mice (Figure 4(b); *F*_2,70_ = 2.318, CA1: *p* = 0.093, CA3: *p* = 0.004, and DG: *p* = 0.931, Figure 4(c); *F*_2,70_ = 5.209, CA1: *p* = 0.242, CA3: *p* < 0.001, and DG: *p* = 0.739).

To examine whether PNNs are associated with PV-positive neurons in the PTZ-kindled mouse hippocampus, we carried out a quantitative analysis (Figures 4(d)–4(g)). In the CA1 and CA3 areas, we found that the percentage of PV-positive neurons not surrounded by both WFA- and Cat-315-positive PNNs was significantly lower in PTZ-kindled mice (Figure 4(d); *F*_2,70_ = 2.164, CA1: *p* = 0.035, CA3: *p* = 0.049, and DG: *p* = 0.637). While there was no significant difference in the percentage of PV neurons surrounded by both WFA- and Cat-315-positive PNNs (Figure 4(e); *F*_2,70_ = 0.693, CA1: *p* = 0.102, CA3: *p* = 0.336, and DG: *p* = 0.997), the percentage of PV-positive neurons surrounded by WFA-positive PNNs was significantly higher in the CA1 region of PTZ-kindled mice (Figure 4(f); *F*_2,70_ = 0.965, CA1: *p* = 0.047, CA3: *p* = 0.34, and DG: *p* = 0.953). There was no significant difference in the percentage of PV neurons surrounded by Cat-315-positive PNNs between control and PTZ-kindled mice in all areas examined (Figure 4(g); *F*_2,70_ = 1.832, CA1: *p* = 0.093, CA3: *p* = 0.057, and DG: *p* = 0.606).

### 3.3. PV-, WFA-, and Cat-315-Positive Fluorescence Intensity in the PTZ-Kindled Mouse Hippocampus

**Figure 5:**
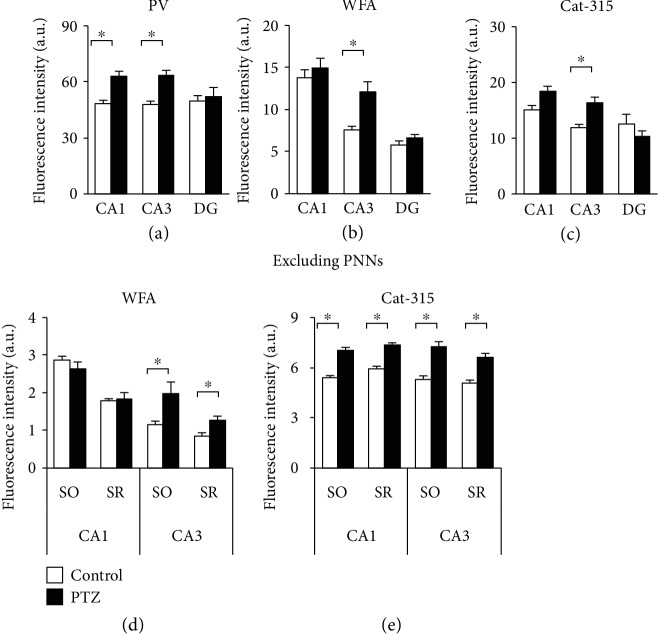
Quantitative analyses of extracellular matrix molecules in the PTZ-kindled mouse hippocampus. Quantified mean fluorescence intensity of PV-positive neurons (a), WFA-positive PNNs (b), and Cat-315-positive PNNs (c), in the mouse hippocampus. Quantified mean fluorescence intensity of WFA-positive molecules (d) and Cat-315-positive molecules, excluding PNNs, in the mouse hippocampus. All data are presented as the mean ± SEM (*n* = 6 mice per group). The *p* values indicate two-way ANOVA followed by Bonferroni *t*-tests. ^∗^*p* < 0.05. PV: parvalbumin; WFA: Wisteria floribunda agglutinin; PNN: perineuronal nets; PTZ: pentylenetetrazole.

To analyze the effect of PTZ kindling on the expression of PV protein, we analyzed the fluorescence intensity of PV-positive neurons in the PTZ-kindled mouse hippocampus (Figure 5(a); *F*_2,351_ = 3.683, CA1: *p* < 0.001, CA3: *p* < 0.001, and DG: *p* = 0.535). Parvalbumin fluorescence intensity was higher in both the CA1 and CA3 areas of the hippocampus in PTZ-kindled mice (Figure 5(a)).

To analyze the effect of PTZ kindling on the expression of WFA- and Cat-315-positive molecules, we analyzed the fluorescence intensity of each WFA- and Cat-315-positive PNN in the PTZ-kindled mouse hippocampus (Figure 5(b); *F*_2,162_ = 1.158, CA1: *p* = 0.521, CA3: *p* = 0.017, and DG: *p* = 0.662, Figure 5(c); *F*_2,270_ = 4.632, CA1: *p* = 0.05, CA3: *p* = 0.006, and DG: *p* = 0.188). We found that WFA and Cat-315 fluorescence intensities were higher only in the CA3 area of the hippocampus in PTZ-kindled mice compared with control mice.

Next, we analyzed WFA- and Cat-315-positive molecules, excluding PNNs, in the PTZ-kindled mouse hippocampus (Figure 5(d); *F*_3,304_ = 5.282, CA1 SO: *p* = 0.252, CA1 SR: *p* = 0.862, CA3 SO: *p* < 0.001, and CA3 SR: *p* = 0.043). In the CA1 area of the hippocampus, the mean fluorescence intensity of WFA-positive molecules, excluding PNNs, was higher in PTZ-kindled mice than in control mice (Figure 5(d)). In both the CA1 and CA3 area of the hippocampus, the mean fluorescence intensity of Cat-315-positive molecules, excluding PNNs, was higher in PTZ-kindled than in control mice (Figure 5(e); *F*_3,304_ = 0.721, CA1 SO: *p* < 0.001, CA1 SR: *p* < 0.001, CA3 SO: *p* < 0.001, and CA3 SR: *p* < 0.001).

### 3.4. GFAP-Positive Astrocytes and iba-1-Positive Microglia in the PTZ-Kindled Mouse Hippocampus

**Figure 6:**
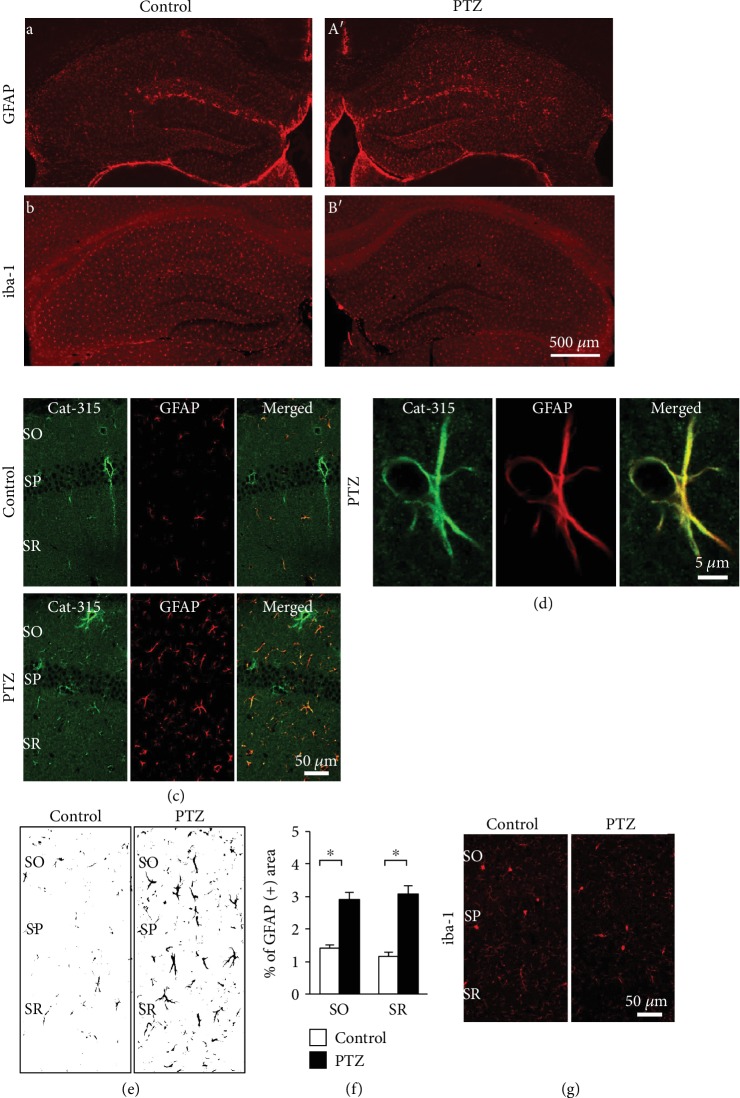
Immunohistochemical analysis of GFAP-positive astrocytes in the hippocampus of PTZ-kindled mice. Representative images of GFAP-positive astrocytes (a, A′) and iba-1-positive microglia (b, B′) in the mouse hippocampus of control (a, b) and PTZ-kindled mice (A′, B′). Representative double immunofluorescent images of Cat-315-positive molecules and GFAP-positive astrocytes in the CA1 (c) of control (upper panels) and PTZ-kindled mice (lower panels). High-magnification double confocal images of Cat-315, GFAP and merged image in the CA1 of the PTZ-kindled mouse (d). Representative converted black and white images used to measure the GFAP-positive area by NIH ImageJ software (e). The percentage of the GFAP-positive area in the SO and SR of the CA1 of vehicle control and PTZ-kindled mice (f). Representative immunofluorescent images of iba-1-positive microglia in the CA1 (g) of control (left panels) and PTZ-kindled mice (right panels). Scale bars: 500 *μ*m in B′ (applies to (a), (b), (A′), and (B′)), 50 *μ*m in (c), 5 *μ*m in (d), and 50 *μ*m in (g). Data are expressed as the mean ± SEM (f; *n* = 6 mice per group). The *p* values indicate two-way ANOVA following by Bonferroni *t*-tests. ^∗^*p* < 0.05. GFAP: glial fibrillary acidic protein; iba-1: ionized calcium-binding adaptor molecule 1; PTZ: pentylenetetrazole; SO: stratum oriens; SR: stratum radiatum.

The effect of PTZ kindling on astrocytes in the hippocampus was also assessed by quantifying GFAP immunoreactivity in the CA1 area (Figure 6(a), (A′), and 6(c)). GFAP-positive astrocytes showed increased ramification (increased branching) in the CA1 area of PTZ-kindled mice compared with controls, indicating astrocytosis (Figures 6(c) and 6(d)). We quantified the area of GFAP-positive signal in the CA1 area (Figures 6(e) and 6(f)) and found that it was significantly higher in PTZ-kindled mice than in control mice (Figure 6(f); *F*_1,44_ = 1.375, SO: *p* < 0.001 and SR: *p* < 0.001). Next, we investigated the relationship between GFAP-positive astrocytes and Cat-315-positive molecules (Figures 6(c) and 6(d)). GFAP-positive astrocytes colocalized with Cat-315-positive molecules in the PTZ-kindled mouse hippocampus (Figure 6(d)).

To examine whether PTZ kindling affects immune activation in the hippocampus, the morphology of iba-1-positive microglia in the CA1 was examined (Figure 6(b), 6(B′), and 6(g)). We found no significant difference in the morphology of iba-1-positive microglia between control and PTZ-kindled mice.

### 3.5. Glutamatergic and GABAergic Synaptic Terminals in the PTZ-Kindled Mouse Hippocampus

**Figure 7:**
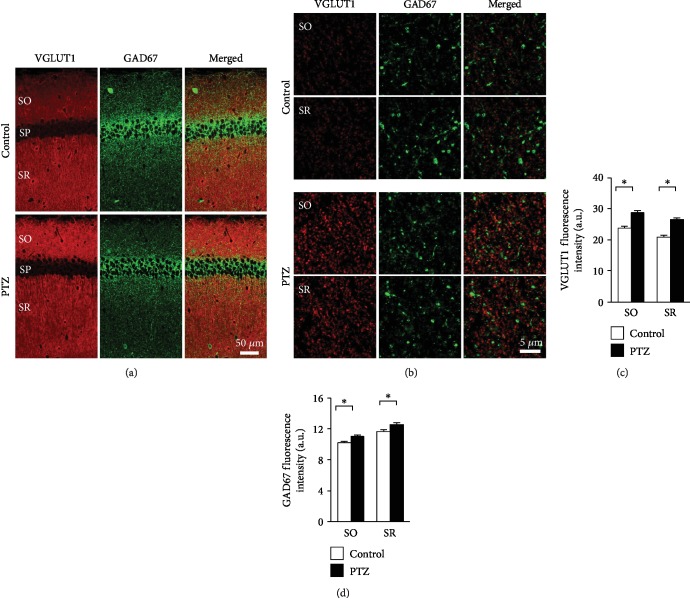
Distribution of VGLUT1-positive and GAD67-positive puncta in the PTZ-kindled mouse hippocampus. Double confocal images of VGLUT1, GAD67, and merged images in CA1 (a) of control (upper panels) and PTZ-kindled mice (lower panels). High-magnification double confocal images of VGLUT1, GAD67, and merged image in the SO and SR of the CA1 (b). Quantified mean fluorescence intensity of VGLUT1-positive puncta (c) and GAD67-positive puncta (d), excluding the GAD67-positive neurons, in the PTZ-kindled mouse hippocampus. Data are expressed as the mean ± SEM (*n* = 6 mice per group). The *p* values indicate two-way ANOVA followed by Bonferroni *t*-tests. ^∗^*p* < 0.05. PTZ: pentylenetetrazole; VGLUT1: vesicular glutamate transporter 1; GAD67: glutamate decarboxylase 67; SO: stratum oriens; SR: stratum radiatum.

To investigate whether synaptic terminals are altered in PTZ-kindled mice, we labeled excitatory (VGLUT1-positive) and inhibitory synaptic terminals (GAD67-positive) (Figures 7(a) and (b)). VGLUT1- and GAD67-positive synaptic terminals were found in the neuropil of the mouse hippocampus (Figure 7(b)). As expected, VGLUT1-positive synaptic terminals did not colocalize with GAD67-positive synaptic terminals (Figure 7(b)). We quantified VGLUT1- and GAD67-positive signal intensities, excluding GAD67-positive neurons, in the hippocampus CA1 area and found that both VGLUT1 and GAD67 fluorescence intensities were higher in PTZ-kindled mice than in control mice (Figure 7(c), *F*_1,236_ = 0.188, SO: *p* < 0.001, SR: *p* < 0.001; Figure 7(d), *F*_1,236_ = 0.022, SO: *p* = 0.002, SR: *p* = 0.001).

## 4. Discussion

The results of this study indicate that molecular components of the ECM, namely, WFA- and Cat-315-positive molecules, increase in the hippocampus of mice after kindling. We also found an increase in activated astrocytes in the hippocampus of PTZ-kindled mice, and we speculate that these activated astrocytes may be responsible for the increase in ECM molecule expression and secretion.

The present study showed an increase in WFA- and Cat-315-positive PNNs in the hippocampus after kindling acquisition. To our knowledge, no one has reported changes in the number of PNNs in PTZ-kindled mice. While previous studies have shown that aggrecan Cat-315-positive molecules increase from day 2 to 7 after status epilepticus in pilocarpine-induced epilepsy model rats [43] and phosphacan-positive PNNs decrease in the kainic acid-induced epilepsy model [44], neither of these studies looked at changes in Cat-315-positive PNNs. Although the results from these studies are not completely consistent with ours, all three studies demonstrate a change in PNNs using different animal models of epilepsy. Differences in the methods, animal species, and stimulation pathways used to induce epilepsy [45] may have contributed to the differences in results. It is widely thought that WFA-positive PNNs control plasticity. However, several reports have suggested the possibility that Cat-315-positive PNNs is involved in plasticity [14, 41]. Aggrecan expression in PNNs signals the end of the critical period [17, 46]. Therefore, this study suggested that the increase in PNNs after kindling decreases neuroplasticity in the hippocampus.

Seizure activity is not randomly spread throughout the brain; it is generated and transmitted by specific anatomical pathways [47]. During development, many studies have shown that PNNs are formed in a stimulus-dependent manner that takes days [15, 48, 49]. Therefore, it makes sense that the number of PNNs increases to maintain the repetitive stimulation pathway caused by PTZ administration. Based on the role of PNNs in synaptic stability and their location around GABAergic interneurons, this structural change may contribute to the progression of epilepsy. The density of both WFA- and Cat-315-positive PNNs was significantly higher only in the CA3 region. The clear reason is unknown. However, the CA3 region is suggested to play a critical role in generation of hippocampal epileptiform activity [50, 51]. Moreover, it has been suggested that the CA3 area is susceptible to hyperexcitability [52].

In contrast, there was no change in the number of PV-positive interneurons after PTZ kindling, similar to results from other studies [44, 53]. In the mouse hippocampus, around 70% of PV-positive neurons are surrounded by WFA- and Cat-315-positive PNNs, which is consistent with our results [54, 55]. Our study is consistent with studies describing PNNs in the hippocampus in the past [54, 56]. In this study, we show that the proportion of PV neurons covered with PNNs increases after kindling acquisition.

According to recent speculation, PNNs in the hippocampus do not turn over after maturation and can help maintain memories [57, 58]. Because PNNs mainly surround PV-positive interneurons, the increase in PNNs can affect the GABAergic circuit [59]. In particular, since PNNs are thought to inhibit synaptic plasticity [14, 17], the decrease in PV-positive neurons not surrounded by PNNs observed in this study indicates a decrease in plasticity in the hippocampus of mice after kindling acquisition. It has long been known that changes to synaptic plasticity are associated with epileptogenesis [6, 7]. For instance, neural circuits that maintain high excitability are caused by changes in synaptic plasticity due to repetitive stimulation in patients with epilepsy and experimental animal models of epilepsy [60–62]. However, the underlying cause of this change in synaptic plasticity is unknown. We speculate that changes to the ECM, and in particular PNNs, may reduce synaptic plasticity in epilepsy.

The extracellular space (ECS) is an important mediator of neuronal plasticity [63]. In this study, the expression of WFA- and Cat-315-positive molecules present in the ECS increased in the PTZ-kindled model. It is unclear whether ECM molecules present outside PNNs have the same functions. ECM molecules in the developing CNS control nerve migration, axonal outgrowth, synapse formation, and synaptic maturation [64–67]. While PNNs are abundantly expressed in the spinal cord, it is not the PNNs but the ECM molecules that suppress axonal extension during regeneration after spinal cord injury [68, 69]. The structure of the net itself is not important since a random collection of ECM molecules has the same effect. Therefore, we propose that the increase in ECM molecule expression in the ECS observed in PTZ-kindled mice decreases synaptic plasticity in the hippocampus.

In mice lacking tenascin-R, which is an extracellular matrix glycoprotein, the progression of kindling due to electrical stimulation is delayed [70]. Furthermore, enzymatic removal of hyaluronan by hyaluronidase treatment reduced kainate-induced hippocampal mossy fiber sprouting, one of the salient features associated with temporal lobe epilepsy [71]. Seizure upregulates the expression of multiple ECM molecules, including tenascin-C, tenascin-R, neuronal pentraxin 2, and hyaluronan [72]. Elevated levels of CSPG, the main component of the brain extracellular matrix, have been seen in patients with temporal lobe epilepsy [73, 74]. Similar to these reports, the results of this study suggest that the ECM is involved in the onset of epilepsy by helping to form and maintain hyperexcitable networks during kindling.

In the present study, we found that astrocytes, but not microglia, were activated in PTZ-kindled mice. Activated astrocytes are observed in the hippocampus of animal models of epilepsy [75, 76] and in the human hippocampus with temporal lobe epilepsy [77]. Reactive astrocytes show increased expression of GFAP and are present in large numbers due to reactive astrocytosis [78, 79]. Astrocytes can increase the volume of the ECS by producing ECM molecules such as CSPG [80, 81]. In addition, astrocytes play a role in synaptic interactions and regulate synaptic strength [82]. Whether astrocyte activation leads to the increase in ECM molecules following PTZ kindling is unknown. It is reasonable to assume that both astrocyte activation and increased ECM molecules may be directly related to the imbalance in excitation and inhibition, as well as changes in synaptic plasticity during epileptogenesis. It has been suggested that the development of drugs that target ECM synthesis and degradation will lead to effective antiepileptic treatments [72]. In addition, our study suggests that therapeutic agents targeting astrocytes may also be effective. Further studies are needed to determine whether inhibiting astrocyte activation suppresses ECM molecule secretion and epilepsy formation.

Seizures are the result of an imbalance of excitation and inhibition caused by excessive excitability and/or imperfect GABAergic circuits. We showed that VGLUT1- and GAD67-positive synaptic terminals increased in PTZ-kindled mice. An increase in VGLUT1 in the hippocampus has also been shown in other animal models of epilepsy, which is consistent with the results of this study [83]. One of the glutamate receptors, the NMDA receptor, is the main focus of research on the molecular mechanisms underlying epileptogenesis. Much evidence indicates that NMDA receptors are involved in the pathogenesis of epilepsy firing [84–86] and in seizure-induced selective excitotoxic cell death in the hippocampus [87, 88].

In this study, we show that the expression of PV protein in PV-positive neurons increased in the hippocampus after PTZ kindling. PV, which is a calcium-binding protein, regulates intracellular calcium dynamics in PV-expressing neurons [89, 90]. In PV-positive neurons of the somatosensory cortex, the expression level of PV protein depends on the input stimulus [91]. PTZ, a noncompetitive GABA_A_ receptor antagonist, induces status epilepticus, a state of increased neural excitation in the hippocampus [92]. Thus, it is possible that the high excitability caused by status epilepticus in PTZ-kindled mice leads to the increase in PV protein expression in the hippocampus. In fact, we found that VGLUT1-positive excitatory synaptic terminals increased in the hippocampus of PTZ-kindled mice. Furthermore, seizures dramatically increase mRNA levels and protein expression of the brain-derived neurotrophic factor (BDNF) both in epilepsy animal models and humans with epilepsy [93–95]. BDNF is known to increase PV protein expression [96, 97], and we speculate that increased BDNF expression increased PV protein expression in this study. Further studies are needed to determine how BDNF expression changes in PTZ-kindled mice.

## 5. Conclusions

We found that astrocyte activation, increased expression of ECM molecules in the ECS region, and increased PNN formation occurred simultaneously in PTZ-kindled epilepsy model mice. We propose that changes in synaptic plasticity due to astrocyte activation and increased ECM molecules may contribute to epileptogenicity.

## Data Availability

The data used to support the findings of this study are included within the article.

## Abbreviations

CNS: Central nervous system
CSPG: Chondroitin sulfate proteoglycans
ECM: Extracellular cellular matrix
PNN: Perineuronal nets
WFA: Wisteria floribunda agglutinin
PV: Parvalbumin
ECS: Extracellular space
PTZ: Pentylenetetrazole
GFAP: Glial fibrillary acidic protein
VGLUT1: Vesicular glutamate transporter 1
GAD67: Glutamate decarboxylase 67
iba-1: Ionized calcium-binding adaptor molecule 1.
